# Enhanced Thermal Conductivities of Liquid Crystal Polyesters from Controlled Structure of Molecular Chains by Introducing Different Dicarboxylic Acid Monomers

**DOI:** 10.34133/2022/9805686

**Published:** 2022-07-16

**Authors:** Xiao Zhong, Kunpeng Ruan, Junwei Gu

**Affiliations:** ^1^Research & Development Institute of Northwestern Polytechnical University, Shenzhen, Guangdong 518057, China; ^2^Shaanxi Key Laboratory of Macromolecular Science and Technology, School of Chemistry and Chemical Engineering, Northwestern Polytechnical University, Xi'an, Shaanxi 710072, China

## Abstract

Enhancing thermal conductivity coefficient (*λ*) of liquid crystal polyesters would further widen their application in electronics and electricals. In this work, a kind of biphenyl-based dihydroxy monomer is synthesized using 4, 4'-biphenyl (BP) and triethylene glycol (TEG) as raw material, which further reacts with three different dicarboxylic acids (succinic acid, p-phenylenediacetic acid, and terephthalic acid, respectively) by melt polycondensation to prepare intrinsically highly thermally conductive poly 4', 4”'-[1, 2-ethanediyl-bis(oxy-2, 1-ethanediyloxy)]-bis(p-hydroxybiphenyl) succinate (PEOS), poly 4', 4”'-[1, 2-ethanediyl-bis(oxy-2, 1-ethanediyloxy)]-bis(p-hydroxybiphenyl) p-phenyldiacetate (PEOP) and poly 4', 4”'-[1, 2-ethanediyl-bis(oxy-2, 1-ethanediyloxy)]-bis(p-hydroxybiphenyl) terephthalate (PEOT), collectively called biphenyl-based liquid crystal polyesters (B-LCPE). The results show that B-LCPE possess the desired molecular structure, exhibit smectic phase in liquid crystal range and semicrystalline polymers at room temperature, and possess excellent intrinsic thermal conductivities, thermal stabilities, and mechanical properties. *λ* of PEOT is 0.51 W/(m·K), significantly exceeds that of polyethylene terephthalate (0.15 W/(m·K)) which has similar molecular structure with PEOT, and also higher than that of PEOS (0.32 W/(m·K)) and PEOP (0.38 W/(m·K)). The corresponding heat resistance index (*T*_HRI_), elasticity modulus, and hardness of PEOT are 174.6°C, 3.6 GPa, and 154.5 MPa, respectively, and also higher than those of PEOS (162.2°C, 1.8 GPa, and 83.4 MPa) and PEOP (171.8°C, 2.3 GPa, and 149.6 MPa).

## 1. Introduction

Polymers inherently possess certain defects in macroscopic and microscopic levels, such as molecular chain entanglement, disordered orientation, holes, and impurities, to cause phonon scattering, further leading to low intrinsic thermal conductivity coefficient (*λ*) [1–3], which makes it difficult to meet better comprehensive performances (especially for thermal conductivity and heat dissipation performance) required by the development tendency of miniature, light and intelligent electronics and electricals [4–6]. The simplest and most convenient way to enhance *λ* values of polymers is to prepare thermally conductive polymer composites by doping thermally conductive fillers [7–9], but the high *λ* values are usually at the expense of mechanical properties, processing properties, density, and cost. In addition, the addition amount of fillers is limited by their inherent characteristics, such as high melting point and poor interfacial compatibility with polymer matrix [10–12]. Meanwhile, when *λ* values of thermally conductive fillers are much higher than that of polymer matrix, the thermal conductivities of the polymer composites increase slowly or even hardly change with the great increase of fillers' *λ* [13–15]. Therefore, the enhancement of intrinsic *λ* for polymer matrix is a key point that needs to be broken through urgently.

Researchers usually control physical structures by optimal design of molecular structures to enhance intrinsic *λ* of polymer matrix [16–18]. One recognized method is to embed liquid crystal units in molecular structure to make molecular chains arrange orderly, so that the heat flow could conduct along the direction of ordered chains, which effectively suppresses phonon scattering and improves thermal conductivity of polymer matrix [19–21].

Intrinsically thermally conductive liquid crystal polymers are generally classified into two categories: thermosetting [22–24] and thermoplastic [25–27] polymers. Lu et al. [28] synthesized a biphenyl-type liquid crystal epoxy monomer from 4, 4'-biphenol (BP) and 6-bromo-1-hexene. Then, a series of thermally conductive liquid crystal epoxy resins (with the maximum *λ* of 0.31 W/(m·K)) were prepared at different curing temperatures using 4, 4'-diaminodiphenyl methane (DDM) as curing agent. Yang et al. [29] obtained a kind of tolane-core thiol-ene-tailed liquid crystal monomer by seven-step reaction with 4-iodophenol and ethyl p-hydroxybenzoate as raw materials. The vertically aligned main-chain end-on liquid crystal elastomer films were prepared by in situ photopolymerization and applying electric field using pentaerythritol tetrakis(3-mercaptopropionate) and glyoxal bis(diallyl acetal) as cross-linkers. The corresponding through-plane *λ* (*λ*_⊥_) and in-plane *λ* (*λ*_| |_) values were 3.56 W/(m·K) and 0.24 W/(m·K), respectively, with the value of *λ*_⊥_/*λ*_| |_ reached 15.0. In our previous works, Gu et al. [30] used 4, 4'-diaminodiphenyl ether and 1, 4-bis(4-aminophenoxy) benzene as diamine monomers, 1, 4-bis(3, 4-dicarboxyphenoxy) benzene dianhydride as dianhydride monomer, and 4-phenylethynylphthalic anhydride as capping agent to synthesize novel kinds of liquid crystal polyimide films with *λ*_| |_ of 2.11 W/(m·K) and *λ*_⊥_ of 0.32 W/(m·K). It is worth mentioning that the ratio of diamine monomers was controlled to change liquid crystal range and further to meet curing temperature. Meanwhile, Gu et al. [31] prepared the main chain intrinsically highly thermally conductive liquid crystal epoxy resin (*λ* of 0.51 W/(m·K)) based on biphenyl liquid crystal units by casting method with BP, triethylene glycol, and epichlorohydrin as main raw materials and DDM as curing agent. Furthermore, a kind of branched-type benzophenanthrene-based discotic liquid crystal epoxy monomer was synthesized with pyrocatechol, 2-allyloxyethanol, and m-chloroperoxybenzoic acid, and a type of co-curing agent was prepared from p-hydroxybenzaldehyde, 9, 10-dihydro-9-oxa-10-phosphaphenanthrene 10-oxide, and amino terminated polysilsesquioxane. Then, intrinsically highly thermally conductive/flame-retardant epoxy resins were also obtained by casting with DDM as curing agent, whose *λ*_⊥_ and *λ*_| |_ were 0.34 W/(m·K) and 1.30 W/(m·K), respectively [32]. However, the diversity of curing agents [33–35] and the uncertainty of curing temperature and time [36–38] all pose great challenges to weather thermosetting liquid crystal polymers could form and maintain locally ordered structures or not.

Compared with thermosetting liquid crystal polymers, thermoplastic liquid crystal polymers are the more ideal objects for research because of their clear structures, repeatability in molding, and recyclability [39–41]. Most industrial thermoplastic liquid crystal polymers are all-aromatic liquid crystal polyesters, which possess advantages of high strength, high modulus, and outstanding melt fluidity. But their intrinsic *λ* values are still low (e.g., the *λ* of Vectra A950 type liquid crystal polyester is only 0.22 W/(m·K)) [42], and a large number of rigid structures in molecular chains inevitably raises the phase transition temperature, which is unfavorable for molding and processing [43–45]. Recently, researchers have devoted themselves to optimizing molecular structures of liquid crystal polyesters to decrease phase transition temperature, and simultaneously to enhance the orderliness of molecular structures in the hope of maximizing intrinsic *λ* of liquid crystal polyesters [46–48]. Wu et al. [49] prepared several biphenyl-type polyesters, which contain flexible methylene units with different length, using BP, phenylsuccinic acid, 3-chloro-1-propanol, 6-chloro-1-hexanol, and 9-bromo-1-nonanol by melt polycondensation. But *λ* values hardly exceed 0.30 W/(m·K). Furthermore, Wu et al. [50] used 6-chloro-1-hexanol as flexible structure to prepare intrinsically thermally conductive biphenyl-type copolyesters with strong *π*-*π* stacking by controlling the ratio of succinic acid to phenylsuccinic acid, thus enhancing *λ* to 0.38 W/(m·K). Kim et al. [51] synthesized biphenyl-based liquid crystal polyesters containing flexible groups by in situ polymerization and injection molding, with BP, dodecanedioic acid, and acetic anhydride as raw materials. Under injection molding stress, the liquid crystal polyesters were able to orientate to form microfibrils, resulting in macroscopic anisotropy in thermal conductivity. The *λ* values along the stress direction exceeded 0.50 W/(m·K), which was attributed to the synergistic effect of liquid crystal units and external force field [52, 53].

**Figure 1:**
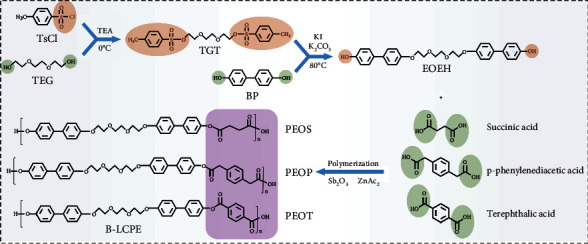
Schematic diagram of synthetic route for B-LCPE.

In this work, biphenyl-based dihydroxy monomer is synthesized using 4, 4′-biphenyl (BP) and triethylene glycol (TEG) as raw material, which further reacts with three different dicarboxylic acids in terms of aliphatic, semiaromatic, and aromatic categories (succinic acid, p-phenylenediacetic acid, and terephthalic acid, respectively) by melt polycondensation to prepare intrinsically highly thermally conductive poly 4′, 4‴-[1, 2-ethanediyl-bis(oxy-2, 1-ethanediyloxy)]-bis(p-hydroxybiphenyl) succinate (PEOS), poly 4′, 4‴-[1, 2-ethanediyl-bis(oxy-2, 1-ethanediyloxy)]-bis(p-hydroxybiphenyl) p-phenyldiacetate (PEOP) and poly 4′, 4‴-[1, 2-ethanediyl-bis(oxy-2, 1-ethanediyloxy)]-bis(p-hydroxybiphenyl) terephthalate (PEOT) followed by casting, called biphenyl-based liquid crystal polyesters (B-LCPE) (Figure 1). The experimental details can be found in Materials and Methods.

## 2. Results and Discussion

Figure S1 shows the ^1^H NMR and ^13^C NMR spectra of intermediate compound (TGT) and dihydroxy monomer (EOEH), and the FT-IR spectra of TGT and EOEH are shown in Figure S2. From Figure S1(a), the characteristic peaks of protons on benzene rings are at 7.5 ppm and 7.8 ppm. The multiple peaks at 3.4 ppm, 3.5 ppm, and 4.1 ppm are from the protons on ethoxy groups. The characteristic peak of protons on methyl group appears at 2.4 ppm. Besides, it can be seen from Figure S1(a1) that the chemical shifts at 128.1 ppm, 130.6 ppm, 132.8 ppm, and 145.4 ppm correspond to the carbon atoms on benzene rings, and the peaks appearing at 68.3 ppm, 70.0 ppm, and 70.4 ppm are attributed to the carbon atoms on ethoxy groups, and the characteristic peak corresponding to the carbon atom on methyl group appears at 21.5 ppm, indicating that molecular structure of the synthesized TGT is consistent with expected design. In Figure S1(b), the characteristic peak appearing at 9.4 ppm corresponds to the proton on hydroxyl group. The characteristic peaks at 6.8 ppm, 7.0 ppm, 7.4 ppm, and 7.5 ppm are attributed to the protons on biphenyl structures, and the peaks appearing at 3.6 ppm, 3.8 ppm, and 4.1 ppm correspond to the chemical shifts of the protons on ethoxy groups. In addition, it can be seen from Figure S1(b1) that the peaks at 115.2 ~ 157.7 ppm are from the carbon atoms on biphenyl structures, and peaks at 67.6 ppm, 69.5 ppm, and 70.4 ppm belong to chemical shifts of carbon atoms on ethoxy groups, indicating the successful synthesis of EOEH. Meanwhile, from Figure S2, compared with TEG, the characteristic peak of -OH at 3356 cm^−1^ does not appear in the spectrum of TGT, while there are abundant absorption peaks appearing at 700~900 cm^−1^ (illustrating the presence of benzene rings in the structure of TGT). The disappearance of hydroxyl groups and the appearance of benzene rings further confirm the successful synthesis of TGT. In addition, compared with BP, EOEH shows stretching vibration peaks of -CH_2_- near 2900 cm^−1^ and absorption peaks attributed to C-O-C at 1000~1300 cm^−1^, further proving that the molecular structure of EOEH is consistent with expected design.

**Figure 2:**
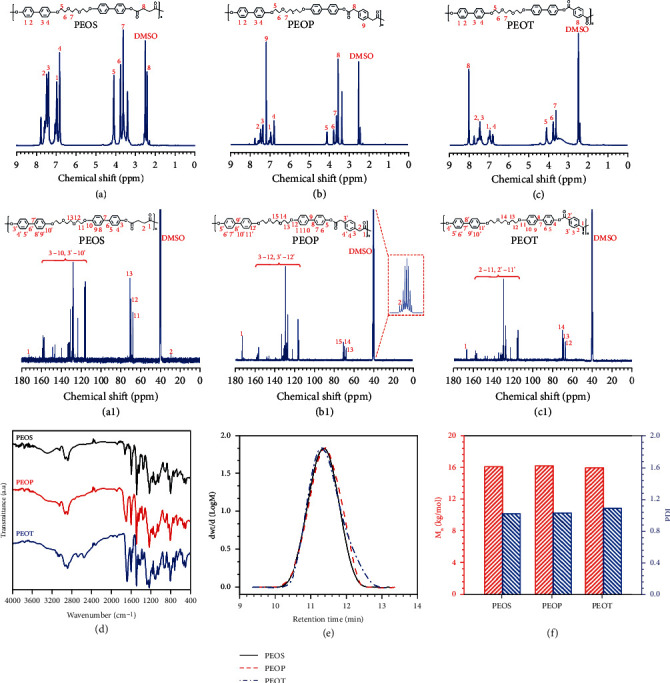
^1^H NMR (a–c), ^13^C NMR (a1–c1), and FT-IR (d) spectra. Molecular weight distribution curves (e) and corresponding values (f) of PEOS, PEOP, and PEOT.

Figure 2 shows the ^1^H NMR, ^13^C NMR, and FT-IR spectra of B-LCPE and presents the corresponding molecular weight and molecular weight distribution of B-LCPE. From ^1^H NMR spectra (Figures 2(a)–2(c)), the characteristic peaks of protons on biphenyl structures are located at 6.8 ppm, 7.0 ppm, 7.4 ppm, and 7.5 ppm, and the multiple peaks observed at 3.6 ppm, 3.8 ppm, and 4.1 ppm are from protons on ethoxy groups, confirming the presence of biphenyl units connected by flexible ethoxy segments in the molecular structure of B-LCPE. Meanwhile, the chemical shift at 2.4 ppm in Figure 2(a) corresponds to protons on methylene groups of PEOS. The characteristic peaks at 3.5 ppm and 7.2 ppm in Figure 2(b) are attributed to the corresponding protons in molecular structure of PEOP. And the peak at 8.0 ppm in Figure 2(c) is the characteristic peak of protons on benzene rings of PEOT. In addition, the characteristic peaks within 110~160 ppm in ^13^C NMR spectra (Figures 2(a1)–2(c1)) belong to carbon atoms in biphenyl structures of B-LCPE, and the peaks appearing in 60~80 ppm are from carbon atoms in ethoxy groups. The chemical shifts of carbon atoms in ester groups of PEOS, PEOP, and PEOT are 172.4 ppm, 173.2 ppm, and 167.2 ppm, respectively, confirming that the molecular structures of B-LCPE are consistent with expected design. In addition, from Figure 2(d), the stretching vibration peaks of C=O in PEOS, PEOP, and PEOT appear at 1734 cm^−1^, 1710 cm^−1^, and 1691 cm^−1^, respectively, and blue shift has taken place compared with the peaks before melt polycondensation (Figure S3(a–c)), indicating the transformation from carboxyl groups to ester groups. From Figure S3(a1–c1), after melt polycondensation, the stretching vibration peaks of C-O in ester groups appear near 1095 cm^−1^, while the new absorption peaks near 755 cm^−1^ (due to the conjugation between ester groups and benzene rings) are from the out-of-plane bending vibration of C-H in benzene rings, which further confirms the existence of ester groups and indicates the success of melt polycondensation. Results of GPC in Figures 2(e)–2(f) show that the number-average molecular weights (M_n_) of PEOS, PEOP, and PEOT are 16.2 kg/mol, 16.3 kg/mol, and 16.1 kg/mol, respectively, all of which are very close to each other and reach prescriptive order of magnitudes for polymers. And polydispersity indexes (PDI) are all less than 1.2, illustrating that B-LCPE have narrow molecular weight distribution, basis of good molding quality, and excellent performances.

**Figure 3:**
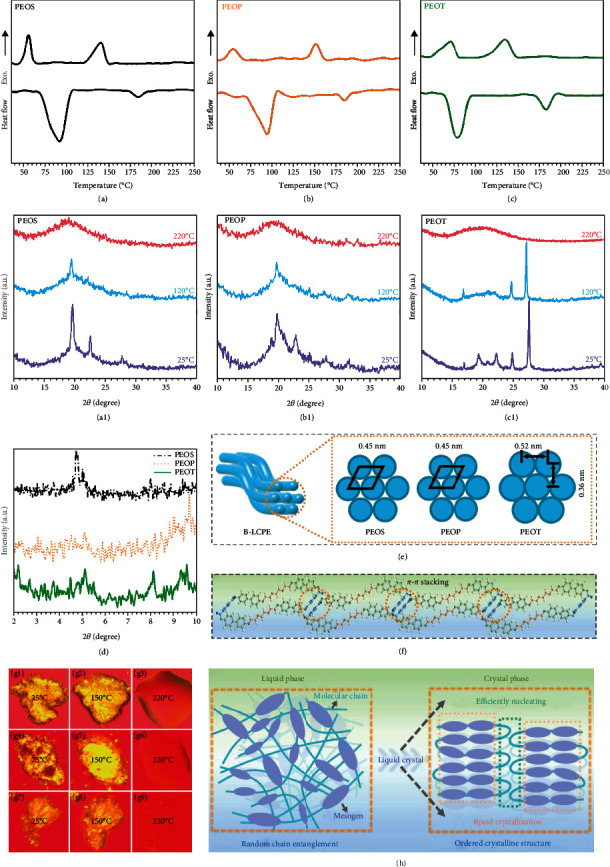
DSC curves (a–c), in situ XRD spectra (a1–c1), SAXRD spectra (d), and proposed intermolecular packing model (e) of B-LCPE; *π*-*π* stacking model of PEOT (f); POM images captured at different temperatures of PEOS (g1–g3), PEOP (g4–g6), and PEOT (g7–g9). Schematic diagram of phase transition process of PEOT (h).

Figures 3(a)–3(c) show the DSC curves of B-LCPE during heating and cooling process, and Figures 3(g1)–3(g9) show the corresponding POM images of heating process. There are two peaks of B-LCPE in heating and cooling process, respectively. During heating process, before the appearance of the first endothermic peak, the observed region under POM shows yellow color (g1, g4, g7), which indicates that B-LCPE are anisotropic and semicrystalline polymers at room temperature. As the temperature increases, the specimen shapes change, and the observed region becomes bright (g2, g5, g8), illustrating that B-LCPE possess both fluidity and anisotropy at this time, revealing liquid crystal characteristics. When the second endothermic peak appears, the color disappears, and the samples become completely transparent (g3, g6, g9), indicating that B-LCPE have turned into isotropic liquid state. Therefore, it can be judged that B-LCPE are thermotropic liquid crystal polymers. The first endothermic peak (the peak temperature is the melting temperature *T*_1_ of crystal) during heating process corresponds to the transition from crystalline state to liquid crystal state, while the second endothermic peak (the peak temperature is the clearing point *T*_2_) corresponds to the transition from liquid crystal state to isotropic liquid state. *T*_1_ for PEOS, PEOP, and PEOT are 93.0°C, 94.3°C and 80.2°C, and the corresponding *T*_2_ are 183.8°C, 185.5°C, and 182.3°C, respectively. Moreover, the DSC results of cooling process further indicate that all B-LCPE are bidirectional thermotropic liquid crystal polymers. As temperature decreases, the first exothermic peak (the peak temperature is denoted by *T*_3_) corresponds to the transition from isotropic liquid state to liquid crystal state, and the second exothermic peak (the peak temperature represents crystallization temperature *T*_4_) corresponds to the transition from liquid crystal state to crystalline state. *T*_3_ of PEOS, PEOP, and PEOT are 140.7°C, 152.2°C, and 135.0°C, respectively, and *T*_4_ in the same order are 57.2°C, 55.0°C, and 72.0°C. It is worth noting that under the same nonisothermal crystallization condition, *T*_4_ of PEOT is significantly higher than that of PEOS and PEOP, proving that PEOT has a relatively strong crystallization ability, which is conducive to the formation of larger-sized crystalline grains. Otherwise, the DSC results of cooling process show great degree of subcooling, which is the normal phenomenon for polymers with viscosity [51, 54].

Liquid crystal behavior and crystallization process of B-LCPE are further investigated by in situ XRD (Figures 3(a1)–3(c1)) during cooling process. At 220°C, the B-LCPE exhibit broad diffused peaks, ascribed to isotropic liquid state currently. As temperature decreases (transforming to liquid crystal state), one or more sharp diffraction peaks appear, revealing the presence of ordered orientation within and between molecular layers. Specifically, PEOS and PEOP show sharp diffraction peaks at 2*θ* = 19.5° (interplanar spacing *d* = 0.45 nm, calculated by Bragg formula) and 19.6° (*d* = 0.45 nm), respectively. And the sharp diffraction peaks of PEOT are located at 2*θ* = 16.9° (*d* = 0.52 nm), 24.6° (*d* = 0.36 nm), and 27.1° (*d* = 0.33 nm), respectively. It can be confirmed that PEOS and PEOP show SmB phase, while the liquid crystal structure of PEOT can be classified as SmF phase [54]. The corresponding intermolecular packing model is shown in Figure 3(e). In addition, the sharp diffraction peak at 2*θ* = 27.1° implies the formation of *π*-*π* stacking structure [46] (Figure 3(f)), because ester group makes contiguous benzene rings shift and offset to form “sidestep” structure, which is conducive to the formation of intermolecular *π*-*π* stacking. As temperature further decreases, crystalline state is formed, while previous sharp diffraction peaks are still present, indicating that the ordered structures formed in liquid crystal state are fixed during crystallization process. On the other hand, new diffraction peaks are observed at 2*θ* = 22.6° (*d* = 0.39 nm) for PEOS, 2*θ* = 22.8° (*d* = 0.39 nm) for PEOP, and 2*θ* = 19.3° (*d* = 0.46 nm), 20.9° (*d* = 0.42 nm), and 22.3° (*d* = 0.40 nm) for PEOT, confirming the occurrence of crystallization process. The diffraction peaks of PEOT are sharper compared with PEOS and PEOP, and the crystallinity of PEOS, PEOP, and PEOT is estimated to be 27.2%, 35.6%, and 67.4% by peak differentiating and imitating by JADE 6 XRD Patterns Processing software [55, 56], indicating that the crystalline grain size and crystallinity of PEOT are relatively larger, which is consistent with DSC results. Figure 3(d) shows SAXRD spectra of B-LCPE. There are sharp diffraction peaks in the range of 2 ~ 10°, further proving the formation of highly ordered layered structure. Figure 3(h) is the schematic diagram for phase transition process of PEOT. The transition from isotropic liquid state to crystalline state is a process from random chain entanglement to ordered crystalline structure. In this process, the presence of liquid crystal state facilitates the crystallization process. Because the packing of biphenyl units during liquid crystal state drives the formation of local ordered domains, which accelerates the crystallization process and promotes the formation of highly ordered crystal, in addition, the strong *π*-*π* stacking interaction of PEOT can effectively promote nucleation [44], which enables PEOT to crystallize rapidly at higher temperature and form highly ordered and well-developed crystal structures.

**Figure 4:**
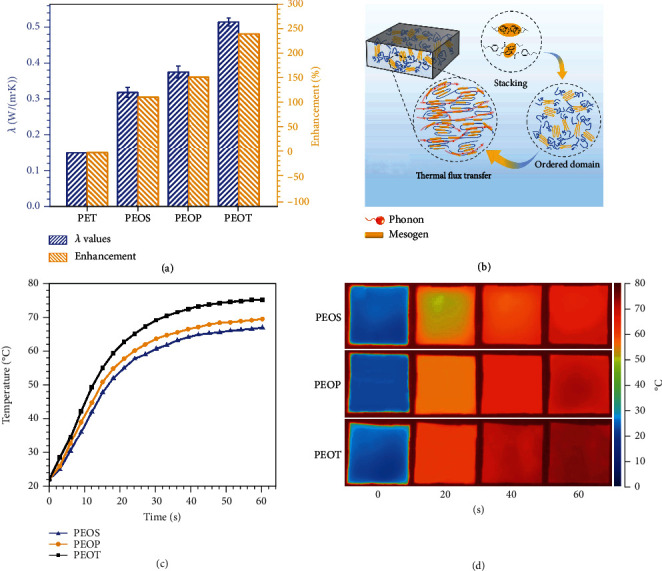
*λ* values of B-LCPE, and enhancement compared with PET (a). Schematic diagram of mesomorphic phase arrangement and thermal flux transfer (b). Relationship curves of temperatures of B-LCPE versus time (c). Infrared thermal images (d) of B-LCPE.

Figure 4(a) shows intrinsic *λ* values of B-LCPE. Compared to that of polyethylene terephthalate (PET, *λ* of 0.15 W/(m·K) [57]), *λ* of PEOS is up to 0.32 W/(m·K) and enhanced by 113.3% due to the introduction of biphenyl liquid crystal units. This is because the biphenyl units stack on the top of each other to form locally ordered domains, which could be fixed during crystallization process, thus benefitting the phonon conduction (Figure 4(b)). However, the intrinsic *λ* of PEOS is still low, due to the large proportion of flexible chain segments in the structural unit, which is not conducive to the stability of crystal structure during crystallization, leading to low crystallinity. The *λ* values of PEOP (0.38 W/(m·K)) and PEOT (0.51 W/(m·K)) which are synthesized after further optimal design of molecular structures are increased by 153.3% and 240.0% compared with that of PET, mainly owing to the further improvement of flexibility for molecular chains, which ensures their kinetically excellent crystallization ability and stability of ordered domains required by thermodynamics, resulting in relatively larger crystallinity. In addition, for PEOT, on one hand, the *π*-*π* stacking structures act like knot, and inhibit the random orientation of molecular chains, and promote the formation of ordered layered domains. On the other hand, the larger crystalline grain size reduces the proportion of amorphous region between one crystalline region and the other, [36] which facilitates the efficient conduction of phonons along ordered domains and endows PEOT excellent thermal conductivity. Figures 4(c) and 4(d) show the infrared thermal images and relationship curves of surface temperatures versus time. It can be seen that the surface temperatures of B-LCPE increase with heating time. PEOT has the highest surface temperature and that of PEOS is the lowest during same heating time. After heating for 60 s, the surface temperature of PEOT reaches 74.9°C, significantly higher than that of PEOP (69.2°C) and PEOS (66.7°C), and the heating rate of PEOT is the largest, confirming that PEOT possesses the optimal thermal conductivity. In addition, Table S1 shows comparison results of *λ* values reported by other works.

**Figure 5:**
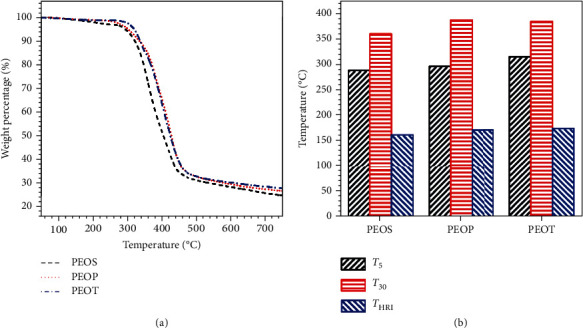
TGA curves (a) and characteristic thermal data (b) of B-LCPE.

Figure 5(a) shows the TGA curves of B-LCPE, and the corresponding characteristic data are shown in Figure 5(b). From Figure 5(a), B-LCPE exhibit similar thermal degradation behavior. The mass losses are within 5% before the temperature reaches 285.0°C, which is mainly attributed to the decomposition of a few of unreacted monomers. The rapid decomposition of B-LCPE in the range of 300~460°C due to the breakage of molecular chains includes the breakdown of ester groups and ether bonds as well as the degradation and carbonization of aromatic rings. The heat resistance index *T*_HRI_ {*T*_HRI_ = 0.49∗[*T*_5_ + 0.6∗(*T*_30_ − *T*_5_)], where *T*_5_ and *T*_30_ are decomposition temperatures with 5% and 30% mass loss, respectively [30]} of PEOT is 174.6°C, which is higher than that of PEOP (171.8°C) and PEOS (162.2°C). In addition, PEOT possesses the highest carbon yield (27.8%). The above proves that PEOT possesses relatively better thermal stability, owing to the largest proportion of rigid structures in molecular structures and the highest crystallinity of PEOT.

**Figure 6:**
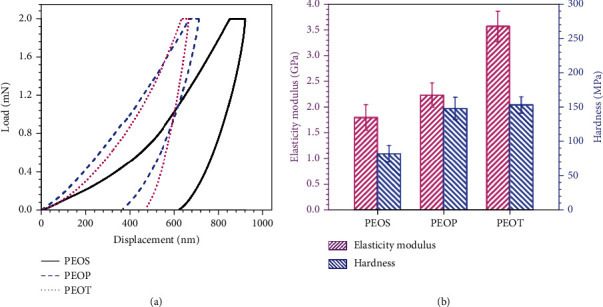
Representative load-displacements curves (a) and elasticity modulus and hardness (b) of B-LCPE.

Figure 6(a) shows the representative load-displacement curves of B-LCPE (the maximum constant load is 2 mN), and the elasticity modulus and hardness data of B-LCPE are shown in Figure 6(b). From Figure 6(a), PEOS, PEOP, and PEOT present excellent indentation resistance with small indentation depth under load. Especially, PEOT exhibits smaller indentation depth and has the optimal indentation resistance. As seen in Figure 6(b), the elasticity modulus and hardness of PEOS, PEOP, and PEOT are 1.8 GPa and 83.4 MPa, 2.3 GPa and 149.6 MPa, and 3.6 GPa and 154.5 MPa, respectively, indicating that PEOT possesses the highest elasticity modulus and hardness. This is because there are larger proportion of rigid structures in molecular chains of PEOT than that of PEOS and PEOP, and the strong *π*-*π* stacking interaction increases intermolecular forces and makes PEOT arrange regularly and compactly at molecular scale, and possess the highest crystallinity, thus endowing PEOT superior mechanical properties.

## 3. Conclusions

B-LCPE possess the desired molecular structure, exhibit smectic phase in liquid crystal range and semicrystalline polymers at room temperature, and possess excellent intrinsic thermal conductivities, thermal stabilities, and mechanical properties. *λ* of PEOT is 0.51 W/(m·K), significantly exceeds that of polyethylene terephthalate (0.15 W/(m·K)), and is higher than that of PEOS (0.32 W/(m·K)) and PEOP (0.38 W/(m·K)). The corresponding heat resistance index (*T*_HRI_), elasticity modulus and hardness of PEOT are 174.6°C, 3.6 GPa, and 154.5 MPa, respectively, and also are higher than those of PEOS (162.2°C, 1.8 GPa, and 83.4 MPa) and PEOP (171.8°C, 2.3 GPa, and 149.6 MPa).

## 4. Materials and Methods

### 4.1. Synthesis of Triethylene Glycol Di(P-Toluenesulfonate) (TGT)

6.3 g of 4-toluene sulfonyl chloride (TsCl) was dissolved in 35.0 mL of dichloromethane (DCM), and 2.0 mL of triethylene glycol (TEG) and 4.6 mL of triethylamine (TEA) were added drop-wise under stirring in ice-water bath. After reacting for 12 hrs, the reaction mixture was washed by saturated Na_2_CO_3_ solution and distilled water for three times, respectively. The organic phase was dried using anhydrous Na_2_SO_4_. DCM was then evaporated with rotary evaporator to obtain yellow liquid. Finally, recrystallization was conducted with ethyl alcohol (EtOH) as solvent, followed by drying in 60°C vacuum oven to obtain TGT (yield: 95.0%).

### 4.2. Synthesis of 4', 4”'-[1, 2-Ethanediyl-Bis(Oxy-2, 1-Ethanediyloxy)]-Bis(P-Hydroxybiphenyl) (EOEH)

6.8 g of TGT and 5.6 g of 4, 4'-biphenyl (BP) were dissolved in 50.0 mL of tetrahydrofuran (THF) followed by magnetically stirring. 20.7 g of K_2_CO_3_ and 3.5 g of KI were added into the above solution, and the mixture reacted at 80°C for 48 hrs. Afterwards, the hot reaction mixture was filtered, while the residue was washed by THF. After repeating for three times, THF in the collected filtrate was removed by rotary evaporator. Then, the residue was washed successively by saturated Na_2_CO_3_ solution, distilled water, EtOH, and DCM, followed by drying in 60°C vacuum oven to obtain EOEH as white solid, namely, biphenyl-based dihydroxyl monomer (yield: 56.0%).

### 4.3. Synthesis and Preparation of B-LCPE

B-LCPE were prepared following the same procedure. Typical synthetic procedure of PEOS was as follows: 14.6 g of EOEH and 3.5 g of succinic acid were loaded into reaction flask equipped with mechanical stirrer. To remove oxygen, the flask was processed by vacuum pumping and nitrogen (N_2_) protection before reaction. Afterwards, 0.3 wt% Sb_2_O_3_ and 0.2 wt% ZnAc_2_ were added into the flask as catalyst. Then, the flask was heated to 160°C in N_2_ atmosphere. When the mixture was homogeneous, the reaction was kept for 2 hrs under magnetic stirring. Meanwhile, the flow rate of N_2_ was increased to blow away by-product. Then, the reaction mixture was heated to 180°C (heating rate was 0.33°C/min) and reacted for 2 hrs. Afterwards, the temperature rose to 200°C and kept for 3 hrs under high vacuum. After that, the reaction mixture was allowed to cool to room temperature under vacuum to obtain tan solid, which was then dried at 60°C under vacuum for overnight (yield: 67.9%). Then, the synthesized B-LCPE were molded by casting method: The solids were added into the mold and heated to 220°C for melting about 20 min and then cooled to room temperature for demolding.

Main materials and Characterizations are detailed in Supplementary Materials.

## Data Availability

The data in this paper cannot be shared at this time as the data also forms part of an ongoing study.

## Additional Points

*Highlights*. (1) Compared with PEOS and PEOP, PEOT has stronger crystallization capacity and higher crystallinity. And the strong intermolecular *π*-*π* stacking of PEOT benefits more ordered orientation of intra- and interlayer molecules. (2) PEOT has high intrinsic *λ* of 0.51 W/(m·K), which is increased by 240.0% than that of polyethylene terephthalate (PET, with *λ* of 0.15 W/(m·K)) and is also higher than that of PEOS (0.32 W/(m·K)) and PEOP (0.38 W/(m·K)). (3) PEOT possesses excellent thermal stability and mechanical properties. The heat resistance index (*T*_HRI_), elasticity modulus and hardness are 174.6°C, 3.6 GPa, and 154.5 MPa, respectively, which are better than those of PEOS (162.2°C, 1.8 GPa, and 83.4 MPa) and PEOP (171.8°C, 2.3 GPa, and 149.6 MPa)

## Supplementary Materials

Supplementary Materials S1 Experimental Section: S1.1 Main Materials; S1.2 Characterizations. S2. Supporting Results: Figure S1: ^1^H NMR (a, b), ^13^C NMR (a1, b1) spectra of TGT and EOEH; Figure S2: FT-IR spectra of TGT and EOEH in comparison with their raw materials; Figure S3: FT-IR spectra of PEOS, PEOP and PEOT in different reaction stages (a-c) and their partial enlargement at fingerprint region (a1-c1); Table S1: Comparison results of *λ* values reported by other works.
