# Supplementary material for: Enhanced Thermal Conductivities of Liquid Crystal Polyesters from Controlled Structure of Molecular Chains by Introducing Different Dicarboxylic Acid Monomers
Source: Research (Wash D C). 2022 Jul 16;2022:9805686. doi: 10.34133/2022/9805686 (PMC9327585; doi:10.34133/2022/9805686)
Supplement: Supplementary Materials — S1 Experimental Section: S1.1 Main Materials; S1.2 Characterizations. S2. Supporting Results: Figure S1: 1H NMR (a, b), 13C NMR (a1, b1) spectra of TGT and EOEH; Figure S2: FT-IR spectra of TGT and EOEH in comparison with their raw materials; Figure S3: FT-IR spectra of PEOS, PEOP and PEOT in different reaction stages (a-c) and their partial enlargement at fingerprint region (a1-c1); Table S1: Comparison results of λ values reported by other works. [file 9805686.f1.docx]

**Supplementary Materials**

**Enhanced Thermal Conductivities of Liquid Crystal Polyesters from Controlled Structure of Molecular Chains by Introducing Different Dicarboxylic Acid Monomers**

Xiao Zhong,^1,2^ Kunpeng Ruan,^1,2^ and Junwei Gu^1,2 *^

^1^ Research & Development Institute of Northwestern Polytechnical University in Shenzhen, Guangdong, 518057, P. R. China.

^2^ Shaanxi Key Laboratory of Macromolecular Science and Technology, School of Chemistry and Chemical Engineering, Northwestern Polytechnical University, Xi’an, Shaanxi, 710072, P. R. China.

Corresponding author, E-mail: gjw@nwpu.edu.cn & nwpugjw@163.com (J. Gu)

**S1. Experimental Section**

**S1.1 Main Materials**

4-toluene sulfonyl chloride, 4, 4’-biphenol, succinic acid, p-phenylenediacetic acid, terephthalic acid, antimony trioxide (Sb_2_O_3_), zinc acetate (ZnAc_2_), triethylamine (TEA) and potassium iodide (KI) were all received from Aladdin Reagent Co., Ltd (Shanghai, China). Triethylene glycol, sodium carbonate (Na_2_CO_3_), sodium sulfate (Na_2_SO_4_) and potassium carbonate (K_2_CO_3_) were all obtained from Macklin Biochemical Technology Co., Ltd (Shanghai, China). Dichloromethane (DCM), ethyl alcohol (EtOH) and tetrahydrofuran (THF) were all purchased from Jinhuada Chemical Reagent Co., Ltd (Guangdong, China).

**S1.2 Characterizations**

^1^H nuclear magnetic resonance (^1^H NMR) and ^13^C nuclear magnetic resonance (^13^C NMR) spectra were recorded on Bruker AV 400 NMR spectrometer (Bruker, Co., Germany) with tetramethylsilane as internal standard and DMSO-*d_6_* as the solvent. Fourier transform infrared (FT-IR) spectra of TEG, TGT, BP and EOEH were captured on Bruker Tensor II type FT-IR equipment (Bruker Optics Inc., USA). FT-IR spectra of other samples were recorded by Bruker Tensor 27 type FT-IR equipment (Bruker Optics Inc., USA). Gel permeation chromatography (GPC) of samples were performed using Waters1515-2414 equipment (Waters Co., USA). *In-situ* X-ray diffraction (*in-situ* XRD) spectra were tested on Rigaku Smartlab type X-ray diffractometer (Rigaku Co., Japan) with scanning speed of 10^°^/min and cooling rate of 10^o^C/min. Small Angle X-ray diffraction (SAXRD) spectra were performed on Rigaku Ultimate IV powder X-ray Cu Ka radiation diffractometer (Rigaku Co., Japan) with scanning speed of 1^°^/min. Thermal gravimetric analyses (TGA) of samples were carried out under argon atmosphere by STA 449F3 (NETZSCH Co., Germany) with heating rate of 20^o^C/min. Differential scanning calorimetry (DSC) curves of the samples were conducted under nitrogen atmosphere with heating rate of 10^o^C/min by DSC1 (Mettler-Toledo Co., Switzerland). Mesomorphic properties were evaluated by WMP-6880 type polarizing microscope (POM, Shanghai Wumo Optical Instrument Co., China) equipped with a hot stage (heating rate of 10^o^C/min). *λ* values of the samples were measured using 7577 F1 probe under block module by Hot Disk TPS2200 thermal constant analyzer (AB Co., Sweden), with the sample size of 20 mm×20 mm×2 mm. Infrared thermal images were captured by Ti 300 infrared thermography (Fluke Co., USA). Mechanical properties of samples were measured using TI980 type nanomechanical test instrument (Bruker-Hysitron Co., USA).

**S2.** **Supporting Results**


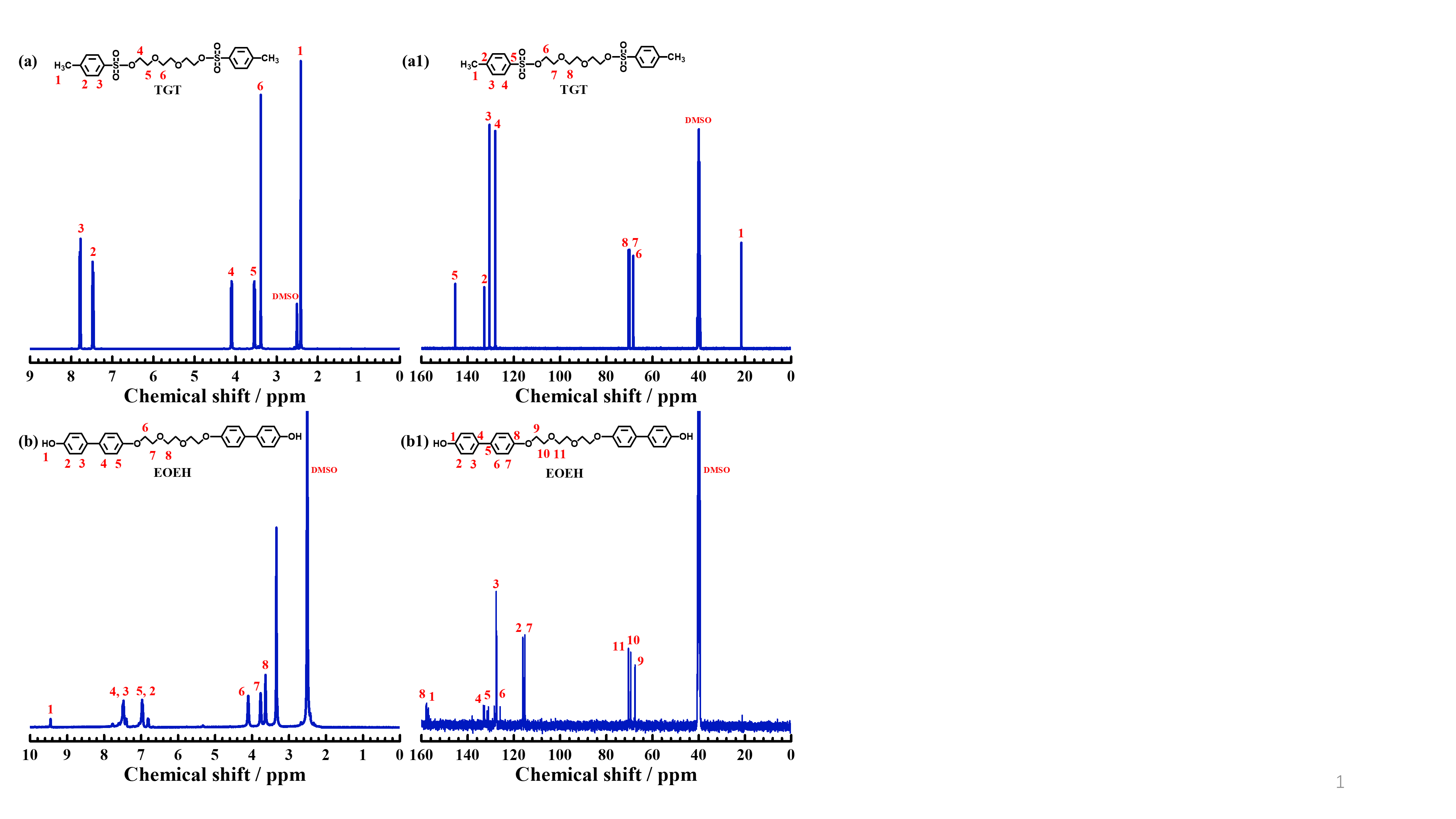


Figure S1: ^1^H NMR (a, b), ^13^C NMR (a1, b1) spectra of TGT and EOEH.


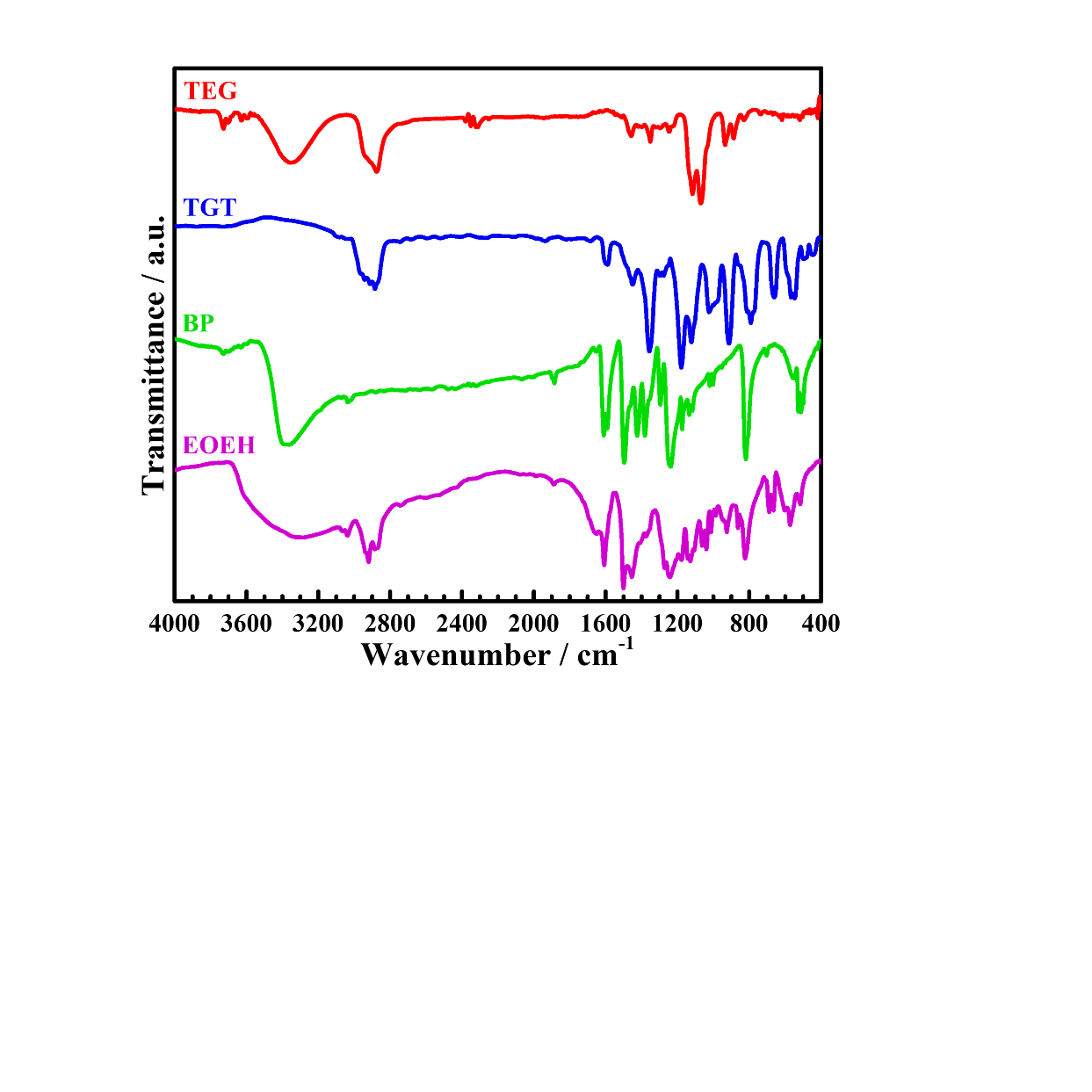


Figure S2: FT-IR spectra of TGT and EOEH in comparison with their raw materials.


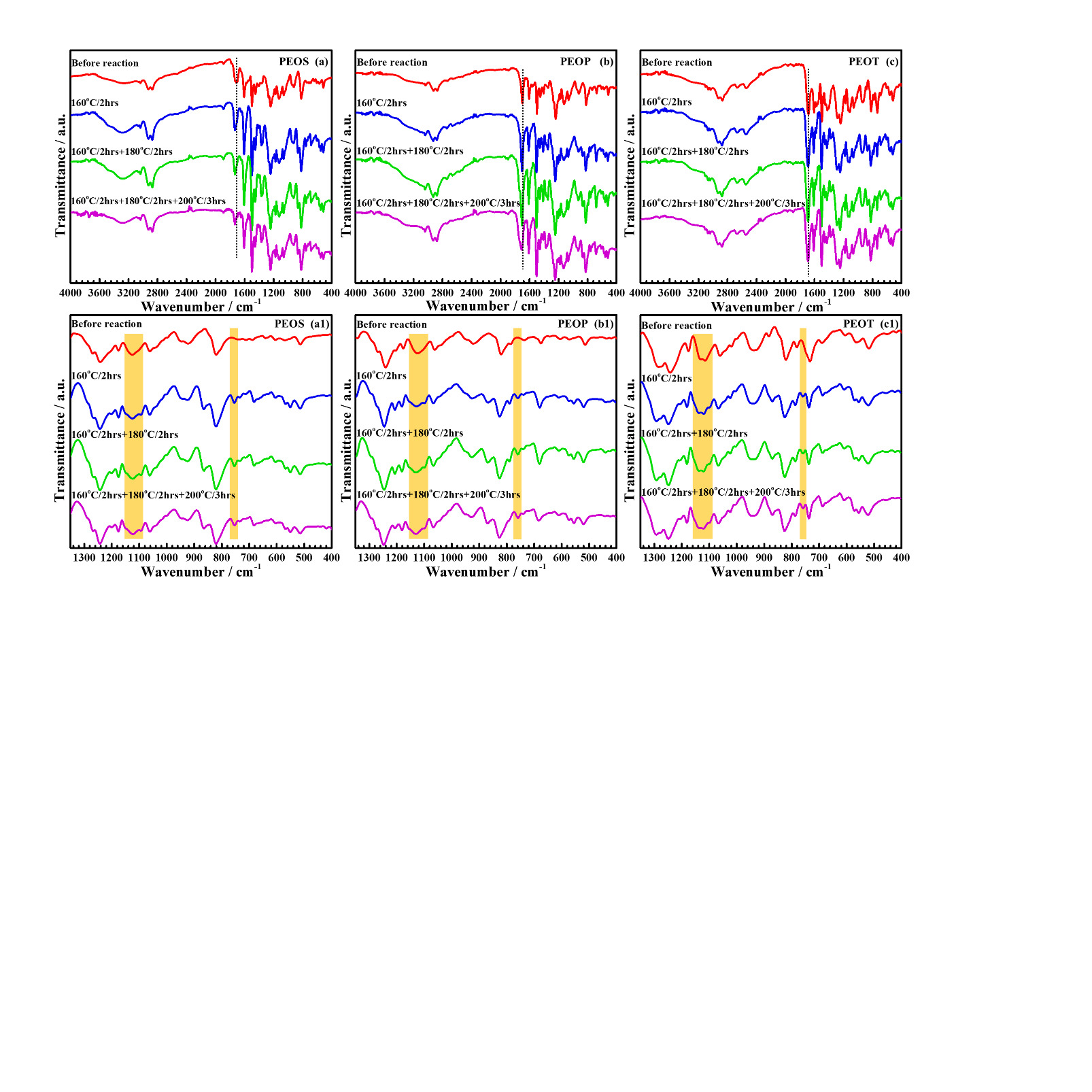


Figure S3: FT-IR spectra of PEOS, PEOP and PEOT in different reaction stages (a-c) and their partial enlargement at fingerprint region (a1-c1).

Table S1: Comparison results of *λ* values reported by other works.


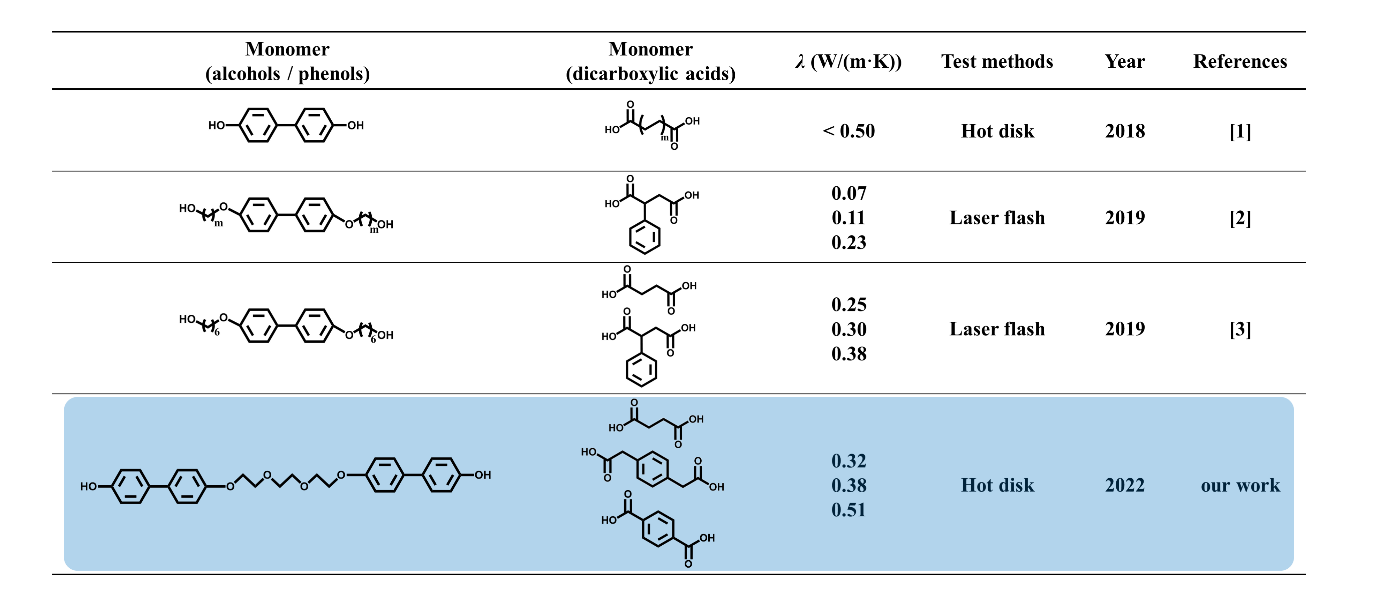


**References**

[1] Y. Kang, Y. Ahn, M. S. Kim et al., "Facile In-situ Polymerization of Thermotropic Liquid Crystalline Polymers as Thermally Conductive Matrix Materials," *Fibers and Polymers*, vol. 19, no. 6, pp. 1143-1149, 2018.

[2] W. Chen, K. Wu, B. Nan et al., "High thermal conductivity and excellent flexibility of crystalline polyesters with flexible segments in main chains," *Reactive and Functional Polymers*, vol. 145, article 104370, 2019.

[3] W. Chen, K. Wu, Z. Qu et al., "Intrinsic high thermal conductive co-polyester based on offset *π*-*π* stacking," *European Polymer Journal*, vol. 121, article 109275, 2019.
